# Genetic Mapping and Diversity of Indigenous and Exotic Rabbits: Adaptive and Conservation Strategies

**DOI:** 10.3390/genes16091050

**Published:** 2025-09-08

**Authors:** Marwa M. Ahmed, Shaymaa M. Abousaad, Soha S. Abdel-Magid, Shoukry M. El-Tantawi, Hatem M. Ali, Essam A. El-Gendy, Nour A. Abouzeid, Lin Yang, Kaliyah Hayes, Mackenzie Skye. Hamilton, Ayman M. Abouzeid, Yongjie Wang

**Affiliations:** 1 Department of Animal Production, National Research Centre, Dokki, Giza 12622, Egypt; m.hassan@nrc.sci.eg (M.M.A.); ss.abdel-magid@nrc.sci.eg (S.S.A.-M.); h.ali@nrc.sci.eg (H.M.A.); 2 Department of Animal Science, College of Agriculture and Environmental Science, North Carolina Agricultural and Technical State University, Greensboro, NC 27411, USA; smabousaad@ncat.edu (S.M.A.); lyang124@aggies.ncat.edu (L.Y.); kahayes1@aggies.ncat.edu (K.H.); 3 Department of Biology, College of Science and Technology, North Carolina Agricultural and Technical State University, Greensboro, NC 27411, USA; 4 Department of Animal Production, Faculty of Agriculture, Cairo University, Giza 12613, Egypt; shtantawey@agr.cu.edu.eg (S.M.E.-T.); essamelgendy@agr.cu.edu.eg (E.A.E.-G.); 5 Department of Biology, College of Arts and Sciences, University of North Carolina at Greensboro, Greensboro, NC 27412, USA; naabouzeid@uncg.edu; 6 Department of Biological Sciences, College of Sciences, North Carolina State University, Raleigh, NC 27695, USA; mhamilt6@ncsu.edu; 7 Department of Agribusiness, Applied Economics and Agriscience Education, College of Agriculture and Environmental Science, North Carolina Agricultural and Technical State University, Greensboro, NC 27411, USA; amabouzeid@ncat.edu

**Keywords:** genetic diversity, conservation, microsatellites, phylogenetics, polymorphism

## Abstract

Background: Climate change threatens global food security, highlighting the need for adaptive traits in livestock to ensure sustainable production. Rabbits, known for their unique adaptability, require the preservation of genetic diversity to maintain resilience. The decline in genetic specificity among indigenous breeds underscores the urgency of conservation efforts to protect these critical resources. Objectives: This study investigates the genetic structure and diversity of indigenous rabbit populations, emphasizing genetic mapping as essential for sustaining adaptability. The findings aim to guide breeding programs that enhance biodiversity and support agricultural resilience. Materials and Methods: This study analyzed both native and exotic rabbit breeds. Native breeds included Black Baladi (BB), White Baladi (WB), Red Baladi (RB), and Jabali (JAB), while exotic breeds included New Zealand White (NZW), American Rex (AR), and Chinchilla (CH). Fourteen microsatellite loci were genotyped in 526 rabbits across all breeds. Results: A total of 467 alleles were identified, with an overall mean of 5.03. The expected heterozygote frequencies were medium to high. Polymorphism was high in BB, JAB, and NZW, and medium in WB, RB, AR, and CH. F_IS_ and F_IT_ values (−0.044 and 0.156) suggested possible non-intensive inbreeding. F_ST_ (0.220) showed breed differentiation and high within-breed variation. The gene flow averaged 1.872, indicating interbreed gene exchange. Neutrality and phylogenetic analyses revealed genetic reshaping; BB, WB, RB, AR, CH, and NZW showed overlap, while JAB retained high specificity. Conclusions: Urgent conservation strategies are essential to preserve native rabbit genetic diversity and unique traits, which are vital for sustaining biodiversity and livestock resilience globally.

## 1. Introduction

Studying the genetic structure of livestock provides animal geneticists with invaluable information on population identification, polymorphism, uniqueness, and genetic diversity. This knowledge enables breeders to improve animal productivity and design conservation programs for genetically unique populations. Although native genetic resources are often lower in productivity, they harbor high genetic variation that can be harnessed to develop strains adapted to harsh environments and resistance to endemic diseases. This has also been demonstrated in Nigeria, where microsatellite analysis of three rabbit populations revealed high levels of polymorphism and greater variation within breeds than between them, highlighting the importance of conserving local rabbit genetic resources for future breeding and sustainability [1]. Consequently, protecting local breeds from potential threats is a priority in sustainable management efforts [2]. For example, a recent microsatellite study in Algeria revealed substantial genetic diversity among native rabbit populations, highlighting the importance of conserving regional breeds as reservoirs of adaptive traits for future sustainability [3].

As climates change and new ecological challenges emerge, conserving and identifying genetic diversity within rabbit populations become increasingly vital. Preserving such unique genetic resources is crucial globally, supporting adaptation to future ecological changes while ensuring food security and sustaining livestock operations across diverse climates and environments.

The genetic structure of rabbits in their native distribution range is the result of many factors, from geographical and ecological, to behavioral and molecular, that hierarchically interact through time and space [4]. Genetic studies so far have focused on a small number of rabbits breeds; therefore, patterns of the population structure of domestic rabbits remain poorly characterized [5].

Microsatellite genotyping allows for the estimation of genetic diversity in breeds and provides additional information for the design and interpretation of breeding programs [6]. In support of this, a genetic evaluation system was developed for New Zealand White rabbits using SSR markers, demonstrating the practical value of microsatellite tools for conservation and germplasm management [7]. Several microsatellite-specific markers have been used to genotype rabbits and to explain population structures. A study reported 257 microsatellite alleles in 12 loci in three rabbit populations [8]. Additionally, it was reported that there was high homogeneity between the native rabbit breeds [9]. Also, the microsatellite genotyping of the native breeds in Egypt revealed moderate to high polymorphism in all loci [10,11].

In Egypt, the genetic resources of native and exotic breeds are maintained in state farms and research institutes, and most of the native rabbits are sustained by smallholders in rural regions. Although the native rabbit breeds naturally adapt to the warm climate, they have not been protected by conservation programs. Accordingly, the total number of does and bucks has become quite small, and some breeds face the threat of losing genetic diversity. The objectives of this study were to explore the genetic structures of the native and exotic rabbit resources in Egypt and to assess the genetic relationship between them.

This study provides a valuable framework for preserving essential genetic traits that contribute to biodiversity and sustainable livestock management. By mapping the genetic diversity and structure of indigenous and exotic rabbit breeds, it offers insights for breeding programs focused on developing resilient breeds that can adapt to specific environmental stressors, such as temperature fluctuations and disease pressures.

On a global scale, this research informs efforts to maintain and enhance the genetic health of rabbit populations, reducing the risks of genetic homogenization and inbreeding. It underscores the importance of genetic resource conservation in supporting resilient ecosystems and sustainable agricultural practices worldwide. By contributing to a broader understanding of genetic diversity in domesticated animals, this study offers essential insights for agricultural and ecological strategies aimed at fostering adaptation and resilience across diverse climates and regions. This study included four of the most common native rabbit breeds in Egypt—Black Baladi (BB), White Baladi (WB), Red Baladi (RB), and Jabali (JAB)—along with three exotic breeds. The Baladi rabbits represent long-established local strains that have adapted to the Egyptian environment, while the Jabali breed is considered an important indigenous genetic resource with strong potential for conservation.

## 2. Materials and Methods

### 2.1. Experimental Animals

This study was carried out in the Department of Animal Production, Faculty of Agriculture, Cairo University, Giza, Egypt. Blood samples were collected, and microsatellite genotyping was subsequently performed.

This study included the four most common native rabbit breeds and three exotic breeds in Egypt. The native breeds were Black Baladi (BB), White Baladi (WB), Red Baladi (RB), and Jabali (JAB), and the exotic breeds were New Zealand White (NZW), American Rex (AR), and Chinchilla (CH). The breeds BB, WB, and RB were formed during the 1970s by the Research Institute of Animal Production (RIAP), Ministry of Agriculture, by a crossbreeding program in three phases. In the first phase, the local random-bred Baladi does, that are spread in the rural regions along the Nile Valley and Delta, were mated by Giant Flemish bucks to form the crossbred population (C1). In the second phase, the crossbred rabbits in C1 were mated by Giant Flander bucks to produce the crossbred population (C2). In the third phase, the individuals of C2 were segregated into three genetic groups by color (black, white, and red) and called BB, WB, and RB, and several generations have been obtained in each group until the breeds stabilized [12]. The JAB rabbits are spread across the western desert in northwest Egypt and Sinai Peninsula in eastern Egypt, and they adapted to the desert environment. The JAB has been maintained by the Desert Research Institute (DRI), Ministry of Agriculture, in a small population [13]. The breed NZW is maintained in RIAP, and the breeds AR and CH are maintained in small populations in RIAP. All native and exotic breeds are randomly bred at the respective research sites. The number of individuals used in this experiment was 127 (BB), 40 (WB), 40 (RB), 112 (JAB), 87 (NZW), 60 (AR), and 60 (CH).

### 2.2. Experiment Procedures

Blood samples (3 mL/individual) were collected from the ear veins of the rabbits in sterilized tubes containing ethylene-di-amine-tetra-acetic-acid (EDTA), and immediately stored at −20 °C. Upon use, the blood samples were thawed, and the genomic DNA was extracted from 200 μL blood according to the protocol of phenol–chloroform extraction. Extracted DNA samples were first visualized on 1% agarose gel. The DNA concentration was estimated at 260 and 280 nm wavelengths using a spectrophotometer (PG Instruments, Lutterworth, UK). The genome samples were genotyped using 14 microsatellite primers (Table 1) [14,15,16,17]. Gradient PCR optimization was performed for all primer sets with annealing temperatures both experimentally optimized, and literature based. PCR was performed in a thermal cycler (Techne, TC3000, Barloworld Scientific Ltd., Stone, UK), using a total volume of 25 µL of the reaction components. The reaction components included 4 µL of genomic DNA (75 ng), 2 µL of each of the forward and reverse primers (25 pmol), 12.5 µL of master mix (Bio Basic Inc., Markham, ON, Canada), and 4.5 µL of PCR-grade water. The PCR program was set at initial denaturation (94 °C/5 min), followed by 35 cycles (denaturation at 94 °C/40 s, annealing at 54–70 °C/40 s, and extension at 72 °C/40–120 s), and ended with a final extension (72 °C/10 min) and final hold (10 s).

The amplified PCR products were separated using 8% non-denatured PAGE. The polyacrylamide gel was prepared (12.8 ml of 30% acrylamide solution, 25.6 mL of ddH_2_O, 800 µL of 10% APS, 40 µL of TEMED, 9.6 mL of 5X-TBE), and 10 ul of each PCR product was loaded into wells, which was validated to yield optimal clear, well-resolved bands for accurate allele scoring. A 50 bp ladder (GeneDireX, Taoyuan, Taiwan, 50 bp DNA ladder RTU) was also loaded to determine the lengths (bp) of the amplified fragments. Electrophoresis was run at 100 v for one hour or until the lower dye escaped from the gel. The gel was submerged in ethidium bromide (Ei.Br) staining solution (0.5 mg ml Ei.Br in 100 mL dH_2_O) for 5–10 min at room temperature. The PCR products of the breeds were visualized and photographed using the WGD-30 WiseDoc Gel Documentation (Daihan Scientific, Co, Ltd., Seoul, Republic of Korea).

### 2.3. Allele Scoring

Allele scoring followed the standard procedures described by [18,19]. After electrophoresis, the PCR products of the breeds were visualized and photographed using the WGD-30 WiseDoc Gel Documentation (Daihan Scientific, Co, Ltd., Seoul, Republic of Korea). Each gel included a 50-bp DNA ladder (GeneDireX) as a molecular size standard. The migration distance of each PCR fragment was compared with the ladder to estimate its size in base pairs. Each distinct band within the expected product size range was treated as one allele. Samples showing a single band were scored as homozygous, whereas those with two distinct bands were scored as heterozygous. Gel images were subsequently processed using bioinformatics TotalLab software [20], which digitized the banding patterns and assigned precise numerical allele sizes (bp). All bands detected were manually validated and confirmed, ensuring that only consistently reproducible signals were considered true alleles, while faint or ambiguous bands were verified through replicated PCR runs, any non-reproducible or artifact bands were excluded, and allele sizing accuracy was confirmed by the standard sizing curve generated by TotalLab software from the DNA ladder, with 99% accuracy (R^2^ ≥ 0.9976). A locus image was randomly selected as a representative example of the genome scanning profile, showing two rabbit breeds: Black Baladi (as a model of native breed) and New Zealand White (as a model of exotic breed) to maintain clarity and conciseness. Once digitized, the gel figures served only as visual confirmation, while the numerical allele size data were compiled across all loci and individuals. As reported in comparable microsatellite studies [21,22], the definitive allele size dataset is presented in a single comprehensive table. Accordingly, the final dataset of allele sizes, which formed the basis for all genetic diversity analyses, is presented in Table 2.

### 2.4. Statistical Analysis

The images were analyzed using TotalLab (v14. 1) [20] and the observed number of alleles (N_o_), effective number of alleles (N_e_), observed heterozygosity (H_o_), expected heterozygosity (H_e_) and polymorphic information content (PIC) were obtained [18,19]. The F-statistics analysis and the neutrality D value were estimated [23,24]. The images of loci Sat3, Sol33, D5Utr4c and D7Utr4a were randomly chosen to draw the phylogenetic charts using TotalLab [20]. The phylogenetic analysis is constructed on a locus basis, so that it can be used with neutrality results to explore the causes of the genetic changes in the breeds during evolution.

## 3. Results

Most of the microsatellite loci were multi-allelic. A total of 467 alleles were recognized in all breeds, and the overall mean was 5.03 alleles/locus/breed. Table 2 presents a summary of the allele information in different microsatellite loci. The mean N_o_ ranged from 2.4 in AR to 9.7 in BB and NZW, and the N_e_ ranged from 2.3 in AR to 7.7 in NZW. The mean difference between N_o_ and N_e_ was large in BB (2.3), JAB (1.1), and NZW (2.0) and small in WB (0.2), RB (0.1), AR (0.1), and CH (0.2). The mean H_o_ ranged from 0.52 in WB and RB to 0.83 in NZW, and the mean H_e_ ranged from 0.46 in AR to 0.82 in NZW. The difference between H_o_ and H_e_ ranged from 0.01 in WB, RB, and NZW to 0.26 in AR. The polymorphism was high (PIC > 0.50) in NZW, BB, and JAB, and was moderate (0.25 < PIC < 0.50) in WB, RB, AR, and CH. The mean inbreeding coefficients (F) were nearly zero in BB (−0.004), WB (+0.030), RB (+0.015), JAB (+0.005), and NZW (−0.044). Inbreeding was evident in AR (F = −0.442) and CH (F = −0.284).

The inbreeding index (F_IS_) in different loci was mostly negative (Table 3), and the mean F_IS_ was nearly zero (−0.044). The variation index (F_IT_) ranged from −0.173 to 0.908. The mean F_IT_ was 0.156 and indicated that the individual variation in rabbit populations accounted for approximately 16%. The mean differentiation index (F_ST_) was 0.220 and indicated high diversification (22%) between the breeds. The rate of gene flow (N_m_) between the breeds was high and ranged from 0.095 to 6.893, with a mean of 1.872.

The mean neutrality (D) values in BB, WB, RB, JAB, NZW, AR, and CH were 0.709, −0.943, −0.628, −0.400, 0.844, −1.074, and −0.489, respectively (Table 4). In BB, the D value at loci Sat5 (1.970), Sol33 (1.770), and D5Utr4e (2.413) deviated from the mean value by 2- to 3-fold. In WB, the D value at locus D5Utr4f (−2.181) deviated from the mean value by about 2.5-fold. In RB, JAB, NZW, AR, and CH, the D values at some loci were similarly deviated from the mean value of the respective breed by 2–4 fold.

**Figure 1:**
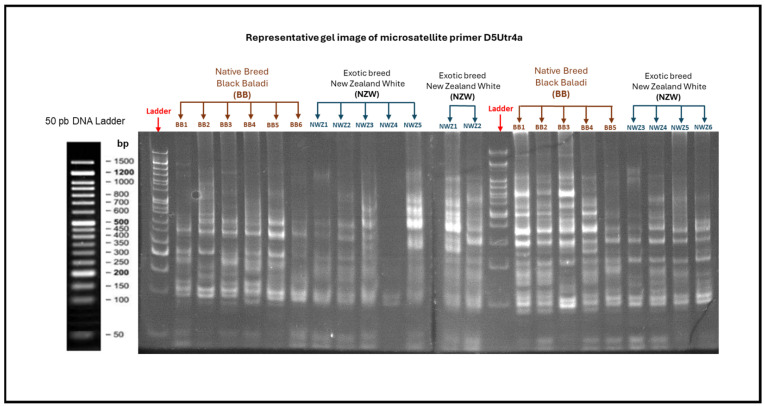
Representative gel image from one microsatellite primer D5Utr4a. This image shows the banding patterns of both a local Egyptian Baladi breed (indigenous) and a New Zealand White breed (exotic), illustrating how alleles were initially visualized on the gel before manual validation and digital scoring.

**Figure 2:**
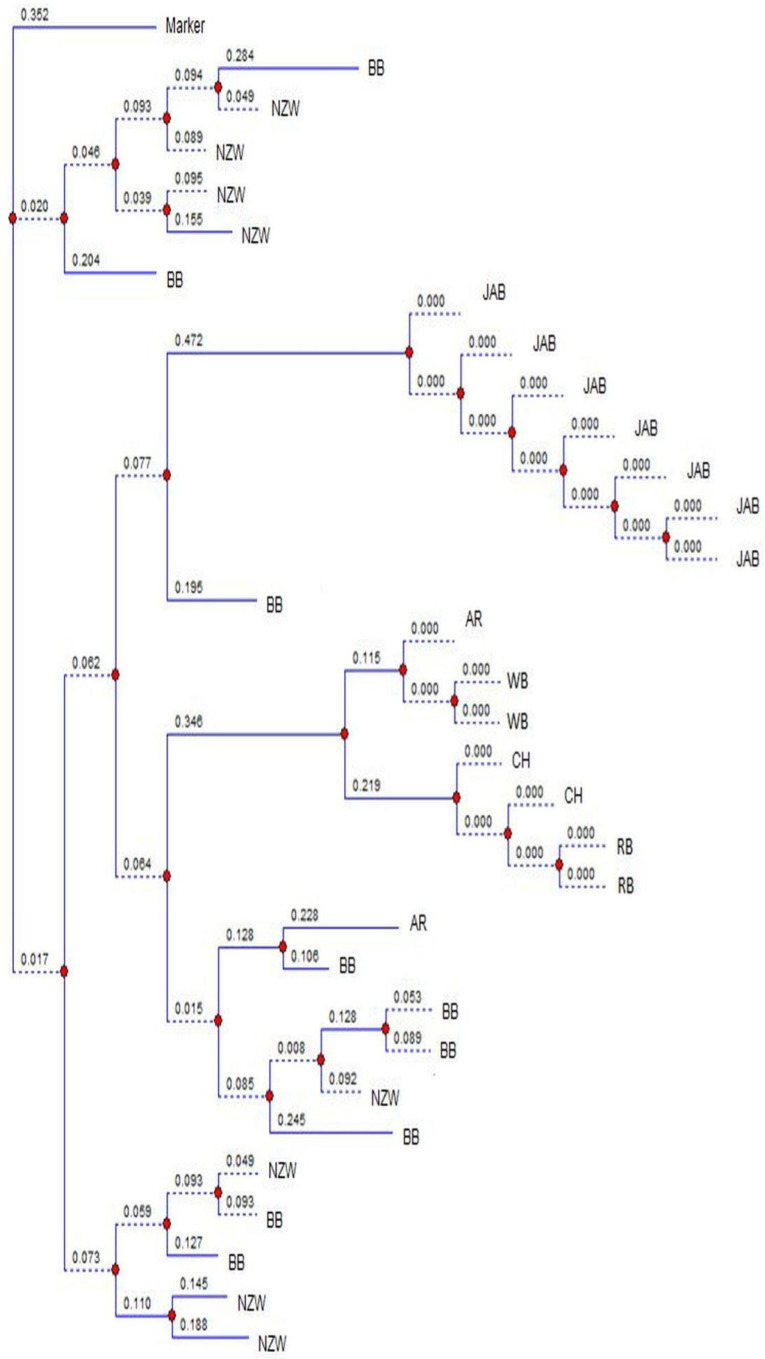
Phylogenetic analysis of the breeds at the microsatellite locus Sat3. BB, WB, RB, JAB, NZW, AR and CH express the breeds Black Baladi, White Baladi, Red Baladi, Jabali, New Zealand White, American Rex and Chinchilla., respectively. Blue lines represent the branches of the dendrogram, red dots indicate divergence nodes, and the numbers on the branches correspond to genetic distance values.

**Figure 3:**
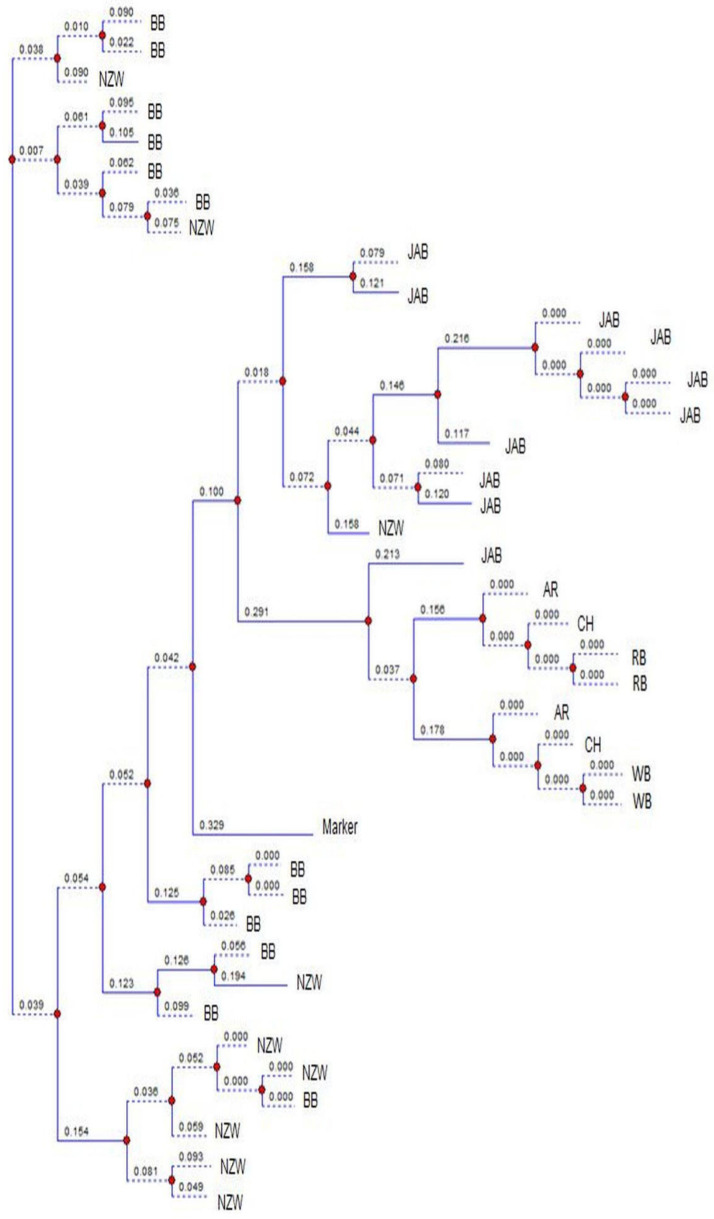
Phylogenetic analysis of the breeds at the microsatellite locus Sol33. BB, WB, RB, JAB, NZW, AR and CH express the breeds Black Baladi, White Baladi, Red Baladi, Jabali, New Zealand White, American Rex and Chinchilla, respectively. Blue lines represent the branches of the dendrogram, red dots indicate divergence nodes, and the numbers on the branches correspond to genetic distance values.

**Figure 4:**
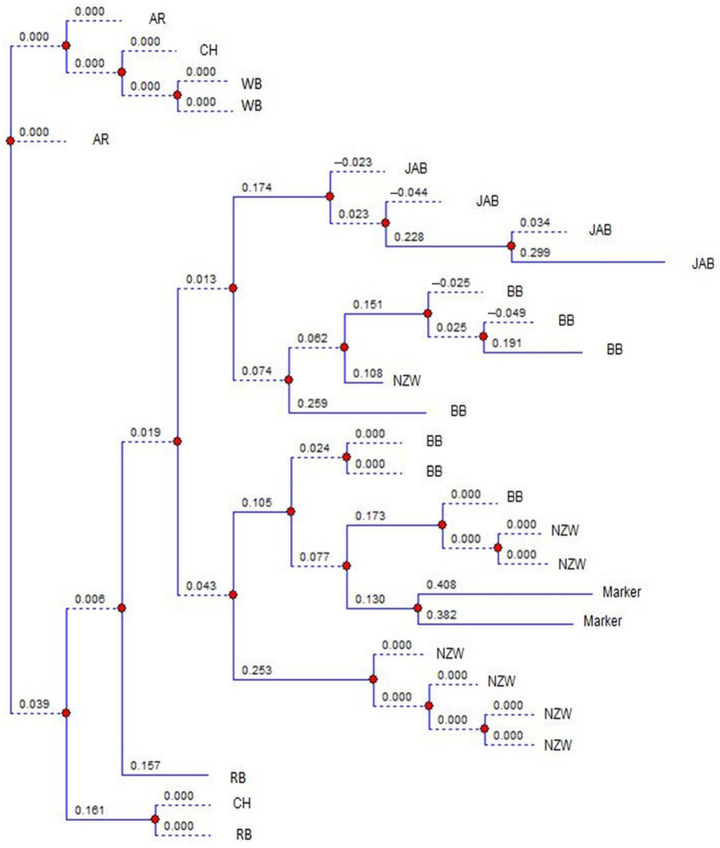
Phylogenetic analysis of the breeds at the microsatellite locus D5Utr4c. BB, WB, RB, JAB, NZW, AR and CH express the breeds Black Baladi, White Baladi, Red Baladi, Jabali, New Zealand White, American Rex and Chinchilla, respectively. Blue lines represent the branches of the dendrogram, red dots indicate divergence nodes, and the numbers on the branches correspond to genetic distance values.

**Figure 5:**
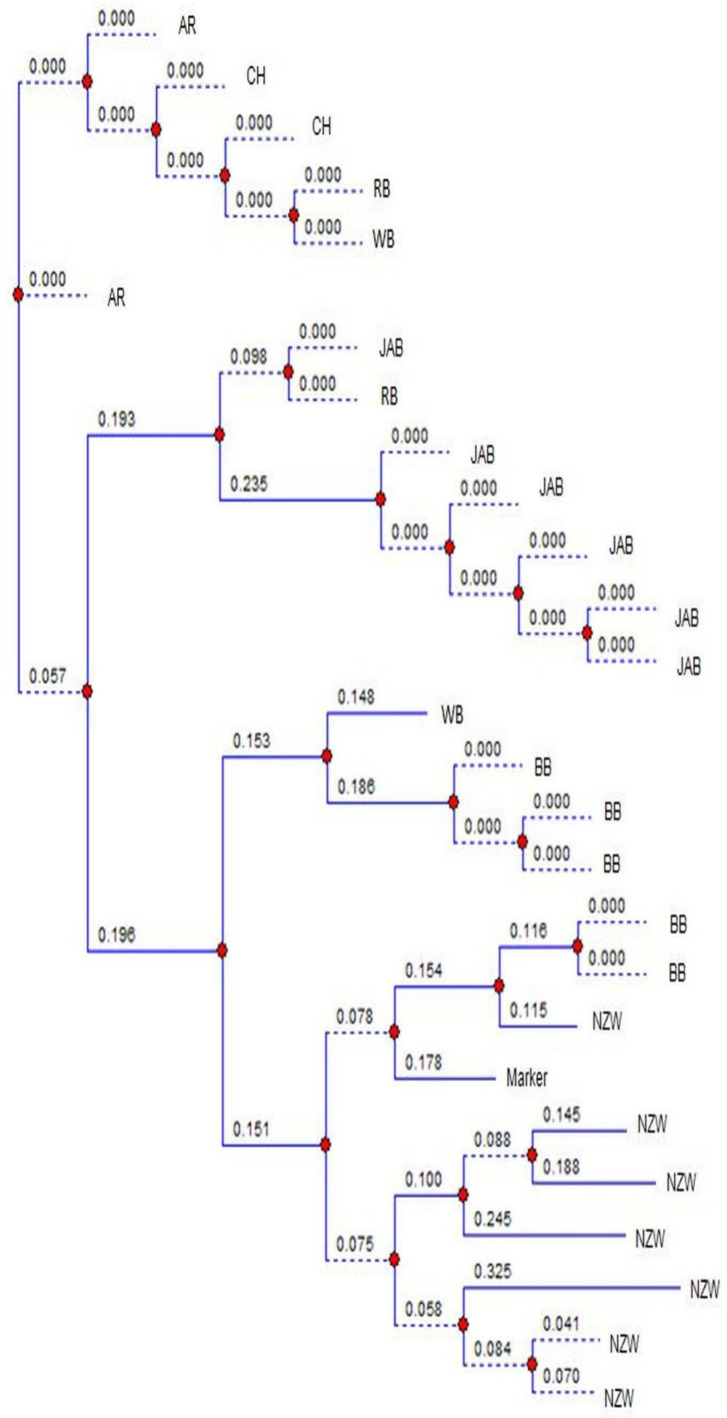
Phylogenetic analysis of the breeds at the microsatellite locus D7Utr4a. BB, WB, RB, JAB, NZW, AR and CH express the breeds Black Baladi, White Baladi, Red Baladi, Jabali, New Zealand White, American Rex and Chinchilla, respectively. Blue lines represent the branches of the dendrogram, red dots indicate divergence nodes, and the numbers on the branches correspond to genetic distance values.

Figure 1 shows the banding patterns of both a local Egyptian Baladi breed (indigenous) and a New Zealand White breed (exotic), illustrating how alleles were initially visualized on the gel before manual validation and digital scoring. Figure 2 presents the phylogenetic tree of locus Sat3. There was an overlap between BB and NZW, and between BB and AR. The phylogenetic tree of locus Sol33 exhibited overlapping between BB and NZW, and between AR and CH (Figure 3). The phylogenetic tree of locus D5Utr4c exhibited overlapping between RB and CH (Figure 4). The phyloge-netic tree of locus D7Utr4a showed an overlap between BB and WB and between WB and RB (Figure 5). The phylogenetic analysis showed that WB and RB formed two separate clusters, and they were the closest to each other, and CH was genetically the closest to them. The breeds WB, RB, AR, and CH have evolved from the same genetic branch. The phylogenetic analysis signaled that two branches were derived from the same genetic node; one branch formed BB and the other branch evolved to WB, RB, AR, and CH. Each of JAB and NZW were derived from a separate genetic branch.

## 4. Discussion

The number of alleles expresses the allele diversity in the breeds. The N_o_ and N_e_ and the difference between them were high in BB, JAB, and NZW, and were small in WB, RB, AR, and CH. The results reveal the allele richness in BB, JAB, and NZW. The values of H_e_ and PIC tended to have similar patterns in the breeds, being medium to high, and were higher in BB, JAB, and NZW than in the other breeds. These results signify the relationship between population size and allele diversity. In BB and NZW, the difference between N_o_ and N_e_ was high and denoted possible changes in the genetic composition. In WB, RB, AR, and CH, low allele diversity was possible since they have been retained in small sizes and restocked by random breeding.

The results are in close agreement with the results obtained for different native and exotic breeds in Egypt and elsewhere. In China, seven native and exotic breeds were genotyped and resulted in 151 alleles in 15 microsatellite loci [25]. The mean N_o_ and N_e_ were 10.07 and 6.625 alleles per locus, respectively. The mean H_e_ ranged from 0.161 in a native breed to 0.889 in an exotic breed, and the mean PIC ranged from 0.625 in a native breed to 0.796 in an exotic breed and indicated high genetic diversity.

A total of 17 microsatellites was used to genotype the Egyptian native breeds BB, JAB, RB, and White Giza (WG) and the exotic line Spanish–New Zealand White (NZW) [26]. The mean number of alleles per locus was 5.41. The mean H_o_ was 0.527 and ranged from 0.477 in NZW to 0.581 in WG, where NZW was the population most differentiated. Another study reported moderate polymorphism (0.25 < PIC < 0.50) in European and NZW breeds [27]. In an earlier study [28], the mean N_o_ was 2.71, 2.29, 2.64, 2.43, and 2.00 in WB, RB, AR, CH, and NZW, respectively.

Also, genotyping 12 microsatellite loci in Soviet Chinchilla (SC) and Californian White (CW) breeds yielded 199 alleles in 12 microsatellite loci with high polymorphism, and the breeds showed low differentiation (F_ST_ = 0.04) [29]. The number of alleles ranged from 4 to 11 in SC and 6 to 10 in CW. In Taiwan, five rabbit populations representing different regions were genotyped by 18 microsatellites [30]. The average values of N_o_, N_e_, H_o_, and H_e_ were 5.50, 2.437, 0.442, and 0.568, respectively, and revealed the inbreeding impact on local rabbits in Taiwan. The F_ST_ between the native rabbit populations was 0.232 and indicated remarkable genetic diversity between the breeds. Our results also coincide with earlier research on Tunisian indigenous rabbit populations, which reported high genetic diversity (Ho = 0.3–0.5) and abundant genetic variation in Tunisian rabbits [31]. Additionally, our findings align with prior research such as [32], who described similar genetic structuring in Egyptian Delta rabbits.

It was reported that if the F_ST_ exceeds 0.15, it suggests a high degree of differentiation [33]. The results in our study provided evidence for breed differentiation (F_ST_ = 0.220). It also indicated that most of the variation is within breeds. The results of F_IS_ and F_IT_ denoted a possible inbreeding history of the breeds. The F values suggested that WB, RB, and JAB have experienced non-intensive inbreeding. The rate of gene flow (N_m_) between the breeds revealed that they have possibly practiced gene exchange by disassortative mating. A significant differentiation (F_ST_ = 0.11) between Tunisian indigenous rabbit populations was previously reported [31]. In China, the native and exotic rabbit breeds had a mean F_ST_ of 0.099 indicating barely fair differentiation between the breeds (10%), and the N_m_ was high (0.818 to 6.031) denoting possible gene exchange between the breeds [31]. The Egyptian native breeds BB, JAB, RB, and White Giza (WG) and the exotic breed NZW had an inbreeding coefficient (F_IT_) of 0.279, and the heterozygote deficit within populations (F) ranged from 0.045 in NZW to 0.266 in BB [26]. The F_ST_ values indicated that NZW was the most differentiated population (F = 0.194). Galal et al. reported low genetic variation in Egyptian native breeds [34].

A neutrality test signifies the past and current genetic state of a population, in terms of random versus non-random evolution. The distribution of D values is naturally conservative, and so the great deviation from the mean D expresses the power of D values in response to the genetic changes in a population [23,35]. In this study, the great deviations of D values, in some loci, from the breed overall means reflect significant evolution events in the breeds. The breed BB had been split from a crossbred population and had not experienced selection. Similarly, NZW is randomly bred over subsequent generations, with no evidence of a breeding history. Therefore, the D values in BB and NZW denote the allele richness, with intermediate frequencies for most of the alleles, and the overlap between BB and NZW was due to disassortative mating. The breeds WB, RB, AR, and CH exhibited negative mean D values, denoting that they have possibly endured genetic drift to evolve to modern breeds and the overlap between them was due to disassortative mating. The breed JAB was initiated from a small population and it had possibly undergone a founder effect to evolve in a separate genetic branch; therefore it is highly conserved.

The analysis of the D values and the phylogenetic trees reveals the extent of the breed specificity. The JAB rabbits formed a unique and stable breed. There was overlapping between all the breeds, except JAB. The BB, WB, RB, NZW, AR, and CH breeds lacked genetic specificity, due to the gene exchange between them. Earlier studies reported genetic mixtures among the native and exotic rabbit breeds in Egypt [26,36]. Also, the genetic distances between WB, RB, NZW, AR, and CH were estimated [28]. The shortest distance was between WB and RB (1.77), and the farthest was between NZW and AR (2.13). The results revealed that CH was genetically the closest to WB and RB. The Jabali breed demonstrated a distinct genetic profile that enriches global rabbit diversity and contributes to the broader effort to maintain livestock genetic resources, which are recognized as a cornerstone for food security, climate change adaptation, and the long-term sustainability of animal agriculture. In line with these findings, the Jabali population should be considered a priority for conservation. Both in situ (on-farm management, selective breeding) and ex situ (gene banks, cryopreservation) strategies are urgently needed to safeguard this genetic heritage for future generations.

## 5. Conclusions

The native rabbit breeds possess high genetic variation, making them highly diverse. However, the retention of these breeds in small flocks has led to inbreeding and gene exchange, resulting in a decline in their genetic specificity. This study underscores the urgent need for sustainable conservation efforts to preserve these valuable rabbit resources, which are important for maintaining genetic diversity and adaptability—qualities that hold global significance in fostering resilient ecosystems, biodiversity, and sustainable agricultural practices worldwide.

## Figures and Tables

**Table 1:** Molecular information of microsatellite primers.

Microsatellite.	Length, Bp	Primer Sequence	A°	Reference
Sat3	F, 21 R, 20	5′ AAGCAAGTGCTGGCTGTGCTC 3′ 5′ TCCTGCCCTTAGCTACGCAC 3′	60	[16] [15]
Sol33	F, 20 R, 24	5′GAAGGCTCTGAGATCTAGAT 3′ 5′GGGCCAATAGGTACTGATCCATGT 3′	55	[17] [15]
Sol44	F, 20 R, 24	5′AGGAAGTGAGGGGAGGTGTT 3′ 5′ATAATGTGCTGCCAAAATAGAAAT 3′	58	[17]
Sat5	F, 20 R, 23	5′GCTTCTGGCTTCAACCTGAC 3′ 5′CTTAGGGTGCAGAATTATAAGAG 3′	56	[16]
D5Utr4a	F, 24 R, 18	5′AAAGTGAGCCTGCAGATGAGAGCA 3′ 5′GGGCGGGGCGGTTACAGT 3	65	[14]
D5Utr4b	F, 20 R, 19	5′CAGCGGTAAGAGTGAGAAAC 3′ 5′TCCCCCATAACAAAAGAGG 3′	60	[14]
D5Utr4c	F, 19 R, 21	5′ GCTCTTGGCTCCTGGTTTC 3′ 5′ AGAGTTCTCCGTCCCTGATGG 3′	60	[14]
D5Utr4d	F, 22 R, 22	5′ GCTGCTTTGGCTCCTAATGTGT 3′ 5′ CTTACCGGGAAATCTCTGACCT 3′	60	[14]
D5Utr4e	F, 17 R, 18	5′ AGGTGGGTGAGGAGACC 3′ 5′ TTGTAATCGGCTCACTAT 3′	65	[14]
D5Utr4f	F, 20 R, 19	5′ CCAGCTGGTAATAGTAGAGA 3′ 5′ AAGGCATTTGTGGAGTGAA 3′	60	[14]
D7Utr4a	F, 22 R, 20	5′ TGCTAATGTGCCCAGAAAGGTA 3′ 5′ GGCATCCCAAAAGGCAGTAT 3′	60	[14]
D7Utr4b	F, 20 R, 17	5′ TAGGCATTTAGGGAGTGAAC 3′ 5′ GGAGGGGGATGGTAGAG 3′	60	[14]
D19Utr4a	F, 22 R, 23	5′ CGACCGTGGGCTCAGAAGAA 3′ 5′ TGTATGTGGGTGTGGGTGTAGAG 3′	70	[14]
D19Utr4b	F, 23 R, 23	5′ TGTATGTGGGTGTGGGTGTAGAG 3′ 5′ TACTGTTGCTTGCTGGGATTTTTA 3′	60	[14]

A° = annealing temperature.

**Table 2:** Summary of the microsatellite allele information (mean ± SE) within rabbit breeds.

Breed	Allele Number		Heterozygosity		PIC	F
	N_o_	N_e_	H_o_	H_e_		
Black Baladi	9.7 ± 1.2	7.4 ± 1.0	0.82 ± 0.08	0.80 ± 0.04	0.69 ± 0.06	−0.004
White Baladi	2.9 ± 0.6	2.7 ± 0.5	0.52 ± 0.08	0.53 ± 0.06	0.45 ± 0.06	+0.030
Red Baladi	2.6 ± 0.4	2.5 ± 0.3	0.52 ± 0.12	0.51 ± 0.07	0.43 ± 0.06	+0.015
Jabali	5.3 ± 1.1	4.2 ± 0.9	0.75 ± 0.10	0.69 ± 0.05	0.63 ± 0.06	+0.005
New Zealand White	9.7 ± 1.3	7.7 ± 1.0	0.83 ± 0.06	0.82 ± 0.04	0.73 ± 0.06	−0.044
American Rex	2.4 ± 0.3	2.3 ± 0.3	0.72 ± 0.11	0.46 ± 0.07	0.39 ± 0.07	−0.442
Chinchilla	2.6 ± 0.4	2.4 ± 0.3	0.61 ± 0.11	0.47 ± 0.07	0.40 ± 0.07	−0.284

N_o_ = number of observed alleles, N_e_ = effective number of alleles, H_o_ and H_e_ = observed and expected heterozygosity, PIC = polymorphic information content, F = inbreeding coefficient.

**Table 3:** F-statistics analysis for the variation within and between rabbit breeds and the rate of gene flow (N_m_).

Microsatellite	F_IS_	F_IT_	F_ST_	N_m_
Sat3	0.132	0.337	0.237	0.805
Sol33	0.018	0.232	0.217	0.902
Sol44	−0.079	−0.027	0.048	4.958
Sat5	0.027	0.090	0.065	3.596
D5Utr4a	−0.420	−0.103	0.223	0.871
D5Utr4b	0.665	0.908	0.725	0.095
D5Utr4c	−0.155	0.166	0.278	0.649
D5Utr4d	−0.693	−0.116	0.341	0.483
D5Utr4e	−0.135	−0.095	0.035	6.893
D5Utr4f	−0.042	0.054	0.092	2.467
D7Utr4a	0.048	0.299	0.264	0.697
D7Utr4b	−0.176	−0.173	0.002	-
D19Utr4a	−0.191	−0.026	0.138	1.562
D19Utr4b	0.383	0.635	0.408	0.363
Mean ± SE	−0.044 ± 0.086	0.156 ± 0.083	0.220 ± 0.051	1.872 ± 0.574

**Table 4:** Levels of neutrality in the rabbit breeds.

.	BB	WB	RB	JAB	NZW	AR	CH
Sat3	0.842	−0.333	−1.000	−0.453	1.894	0.364	0.000
Sol33	1.770	−0.333	−1.000	0.119	0.494	0.000	0.667
Sol44	0.059	−0.636	1.080	−0.791	0.273	−1.636	−1.636
Sat5	0.970	−1.636	−0.448	−0.227	1.207	−1.636	−1.636
D5Utr4a	0.794	−1.333	−1.333	−1.386	0.767	−1.333	−1.333
D5Utr4b	−0.847	−1.000	−1.000	-	−0.229	−1.000	−1.000
D5Utr4c	0.755	−1.333	−1.636	−1.333	−0.099	−1.333	−0.636
D5Utr4d	−1.006	−1.333	−1.333	-	−0.606	−1.333	−1.333
D5Utr4e	2.413	−0.636	−0.636	-	2.545	−1.333	−0.636
D5Utr4f	0.896	−2.181	-	-	2.335	−2.944	1.551
D7Utr4a	−0.144	−0.333	0.667	−1.152	0.351	−1.333	−0.333
D7Utr4b	0.807	−1.449	−0.190	1.054	0.028	−2.190	0.810
D19Utr4a	0.912	−1.333	−1.333	0.571	1.912	0.667	−1.333
D19Utr4b	−0.301	0.667	0.000	-	−0.053	−1.000	−1.000
Mean	0.709	−0.943	−0.628	−0.400	0.844	−1.074	−0.489
SE	0.267	0.193	0.228	0.270	0.278	0.295	0.300

BB, WB, RB, JAB, NZW, AR, and CH express the breeds Black Baladi, White Baladi, Red Baladi, Jabali, New Zealand White, American Rex, and Chinchilla.

## Data Availability

The data supporting the findings of this study are available from the corresponding author upon reasonable request.

## Abbreviations

The following abbreviations are used in this manuscript:

BB	Black Baladi
WB	White Baladi
RB	Red Baladi
JAB	Jabali
NZW	New Zealand White
AR	American Rex
CH	Chinchilla
RIAP	Research Institute of Animal Production
DRI	Desert Research Institute
H_o_	Observed Heterozygosity
H_e_	Expected heterozygosity
PIC	Polymorphic information content
F_IS_	Inbreeding index
F_IT_	Variation index
F_ST_	Differentiation index
C1	First Crossbred Population (Baladi × Giant Flemish)
C2	Second Crossbred Population (C1 × Giant Flander)
